# Supplementary material for: Characterization of the Humoral Immune Response to Porcine Epidemic Diarrhea Virus Infection under Experimental and Field Conditions Using an AlphaLISA Platform
Source: Pathogens. 2020 Mar 21;9(3):233. doi: 10.3390/pathogens9030233 (PMC7157568; doi:10.3390/pathogens9030233)
Supplement: Supplementary file 1 [file pathogens-09-00233-s001.pdf]

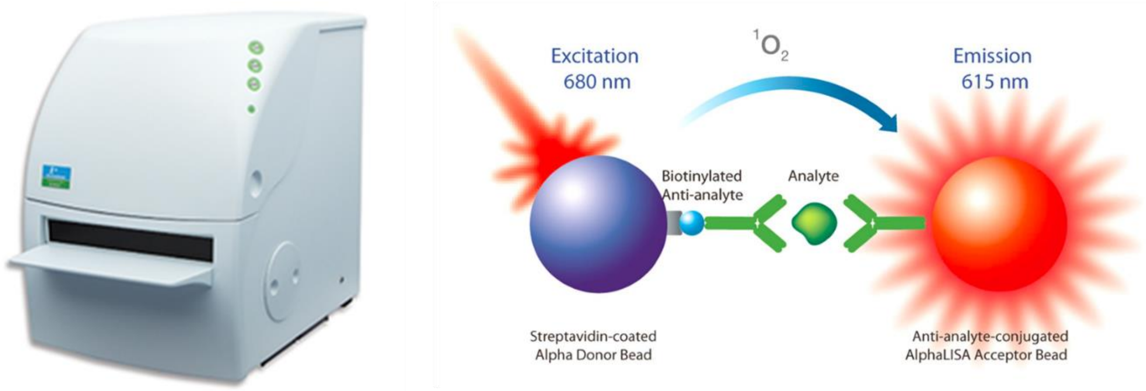

Figure S1. Overall principle of the AlphaLISA assay technology, and image of the EnVision® 2105 multimode microplate reader used in this study.
